# Renaming Schizophrenia and Stigma Reduction: A Cross-Sectional Study of Nursing Students in Taiwan

**DOI:** 10.3390/ijerph19063563

**Published:** 2022-03-17

**Authors:** Yi-Hang Chiu, Meei-Ying Kao, Kah Kheng Goh, Cheng-Yu Lu, Mong-Liang Lu

**Affiliations:** 1 Department of Psychiatry and Psychiatric Research Center, Wan Fang Hospital, Taipei Medical University, Taipei 116, Taiwan; chiuyihang@gmail.com (Y.-H.C.); havicson@gmail.com (K.K.G.); 2 Graduate Institute of Humanities in Medicine, Taipei Medical University, Taipei 106, Taiwan; mykao@tmu.edu.tw; 3 Department of Psychiatry, School of Medicine, College of Medicine, Taipei Medical University, Taipei 106, Taiwan; 4 Psychology of Mental Health Programme, School of Health in Social Science, College of Arts, Humanities and Social Sciences, University of Edinburgh, Edinburgh EH8 9AG, UK; yaya880427@gmail.com

**Keywords:** renaming, stigma, social distance, schizophrenia, nursing students

## Abstract

Schizophrenia is one of the most stigmatized mental disorders. In 2014, schizophrenia was renamed in Mandarin in Taiwan, from the old name of “mind-splitting disease” to new name “disorder with dysfunction of thought and perception”, in an attempt to reduce the stigmatization of schizophrenia. This cross-sectional study aimed to investigate the effects of renaming schizophrenia on its stigma in nursing students. We examined the public stigma, self-stigma, and social distance associated with schizophrenia and compared them before and after the renaming. Basic demographic data and previous contact experience were collected, and participants completed a modified Attribution Questionnaire, the Perceived Psychiatric Stigma Scale, and modified Social Distance Scale. The final sample comprised 99 participants. Assessment revealed that the renaming significantly reduced public stigma, self-stigma, and social distance. Regarding the old and new names for schizophrenia, the fourth-year nursing students scored significantly higher on public stigma and self-stigma than did the first-year students. Personal exposure to individuals diagnosed with mental disorders reduced public stigma toward schizophrenia. The study findings suggest that the renaming of schizophrenia reduced its associated stigma. Providing accurate information, instruction by qualified tutors, as well as exposure to patients in acute exacerbation in hospital settings and recovered patients in the community are important. Further studies with longitudinal design, participants from diverse backgrounds, and larger sample sizes to investigate the effect of renaming on the stigma toward schizophrenia are warranted.

## 1. Introduction

The word stigma is derived from a clutch of Ancient Greek words referring to a symbol deliberately marked on people with socially unacceptable morals or actions. Stigmatization occurs when a person behaves abnormally by societal standards. Stigma and the process of stigmatization comprise the recognition of the distinguishing mark and the subsequent devaluation of the individual [1]. Stigmatization not only affects victims’ behavior but also their faith and emotions. Public stigma refers to the reactions of the general population toward a stigmatized group, whereas self-stigma refers to the internalization of the ideas and the reactions of those affected by a stigma [2,3]. Stigma toward schizophrenia produces deleterious consequences, including lower self-esteem, increased social isolation, hindered search for and adherence to treatment, and reduced access to social support [4].

Schizophrenia is the mental disorder most strongly associated with stereotyping, prejudice, and discrimination [5]. The stigmatization of patients with schizophrenia is common across various countries and cultures [6,7]. The perception that those with schizophrenia are unpredictable and dangerous shapes the public desire for social distance toward these individuals [2]. A cross-sectional study conducted in Taiwan revealed that schizophrenia is associated with the highest degree of stigma among all mental disorders [8].

In recent years, the names of schizophrenia in the local languages of several Asian countries, namely Japan, South Korea, and Taiwan, have been changed [9]. A systematic review found that the renaming of this disorder may be associated with improvements in attitudes toward patients with schizophrenia and with increases in diagnosis announcements [10].

The old name for schizophrenia in Mandarin in Taiwan was based on the concept that it was a “mind-splitting disease,” giving an impression of danger, uncertainty, severity, and the impossibility of recovery. In October 2012, “disorder with dysfunction of thought and perception” was introduced by the Taiwanese Society of Psychiatry as the new name for schizophrenia [9]. This new name reflects the two main clinical characteristics of schizophrenia, cognitive and perceptual dysfunction, and the word “disorder” connotes treatability and the possibility of recovery [11]. However, a text mining analysis of newspaper articles in Taiwan detected no significant difference in negative word usage between articles using the old and new names [12]. A cross-sectional survey to medical students in Taiwan showed that renaming could reduce the stigma and social distance toward people with schizophrenia [13].

Most nursing students will eventually become frontline healthcare professionals and may encounter patients with mental illness including schizophrenia. Prejudice or discrimination in these personnel may influence the quality of the service that they provide. Several studies reported that nursing students regard mental health nursing as the least favorable career option [14,15]. Nursing education and clinical placement carry great duties in shaping the attitudes towards people with mental illness and choosing mental health nursing as careers in nursing students [5]. Expansion of theoretical training, appropriate clinical placement, and more contact experience with people with mental disorders have been proposed to reduce their stigmatization among nursing students [16,17,18]. Other studies, however, found contradicting results for which clinical placement might induce higher levels of stigma toward mental illness in nursing students [5,19].

To the best of our knowledge, the present study is the first to investigate the destigmatizing effect of renaming schizophrenia among nursing students in Taiwan. This study had the following objectives: (1) to evaluate the effect of renaming schizophrenia on stigma reduction among nursing students; (2) to evaluate the effect of psychiatric training in nursing education on destigmatization; and (3) to investigate the potential influential factors on the reduction in stigma.

## 2. Methods

### 2.1. Participants

A cross-sectional study was performed to investigate the effects of renaming on nursing students’ stigma toward schizophrenia in three different dimensions, namely public stigma, self-stigma, and social distance. We also compared the influence of psychiatric education and clinical experience on the stigmatizing attitudes. The survey was carried out on nursing students in the School of Nursing of the Taipei Medical University, Taiwan, who attended their first year and their fourth year of classes from October 2014 to February 2015. After the introduction of the purpose and process of our study at a class meeting, students were invited to participate in our study voluntarily. During the introduction, we illustrated the background information of renaming schizophrenia in Taiwan. The participants were aware that the two names of schizophrenia actually denoted the same condition.

In Taiwan, nursing is a four-year undergraduate degree. Psychiatric training is provided in the fourth year and consists of clinical lectures (on clinical characteristics of mental disorders, their biopsychosocial treatments, and mental healthcare organization), tutorial clinical workshops, and attendance of clinical facilities. Therefore, the fourth-year nursing students who participated in this survey had already received their undergraduate training in psychiatry. By contrast, the first-year nursing students had not received any psychiatric training.

### 2.2. Ethical Considerations

The study protocol was approved by the Institutional Review Board of Taipei Medical University (approval number: 201312002). The Institutional Review Board of Taipei Medical University waived the requirement for the investigators to obtain written consents form for all participants. We informed the students of the study’s purpose, methodology, and relevance prior to commencement. Furthermore, we assured the students that no negative outcomes would occur if they chose not to participate. Confidentiality and privacy of the participants were ensured using an anonymous questionnaire.

### 2.3. Tools

The participants were assessed through filling out the self-administered questionnaires. The first section comprised questions on demographic characteristics (e.g., age, sex, university year, and religion) and previous contact experiences with individuals with mental illness. The second through fourth sections comprised questionnaires on public stigma, self-stigma, and social distance.

#### 2.3.1. Public Stigma

To assess public stigma toward schizophrenia, we used a modified version of the Attribution Questionnaire [20]. Owing to the interitem similarity after translation into Chinese, we extracted 20 items according to experts’ opinions for this study. Compared with the 27-item Attribution Questionnaire, items 4, 12, 19, 21, 22, 24, and 26 were excluded. These 20 items were grouped into nine subscales, namely blame (e.g., I would think that it was Harry’s own fault that he is in the present condition); anger (e.g., I would feel aggravated by Harry); pity (e.g., I would feel pity for Harry); help (e.g., how likely is it that you would help Harry?); dangerousness (e.g., I would feel unsafe around Harry); fear (e.g., Harry would terrify me); avoidance (e.g., if I were an employer, I would interview Harry for a job); segregation (e.g., I think Harry poses a risk to his neighbors unless he is hospitalized); and coercion (e.g., if I were in charge of Harry’s treatment, I would require him to take his medication). Responses were scored on a nine-point Likert scale, ranging from 1 (absolutely not) to 9 (absolutely). Parts one and two of the questionnaire were identical in content except for the fact that one used the new name for schizophrenia and the other used the old name. Items related to pity, help, and avoidance were reversely scored. A composite measure of public stigma is derived by totaling the sum of all statements (range: 20–180). The higher the score, the more discrimination and stigmatization demonstrated. Cronbach’s α = 0.83 in the old name version and Cronbach’s α = 0.82 in the new name version.

#### 2.3.2. Self-Stigma

We assessed self-stigma by using the Perceived Psychiatric Stigma Scale [21]. Taiwanese society emphasizes family and marriage over equal treatment in employment, which is a more common focus in Western society. Therefore, the developers incorporated the items related to family and marriage in the Perceived Psychiatric Stigma Scale. Thus, the use of this scale was considered suitable for examining stigmatization in Taiwan. The questions were formulated in the first person, and subjunctive sentences were used to gauge the respondents’ feelings and opinions. In the first subscale (social ostracism), the respondents were asked to imagine themselves as patients with schizophrenia and presume how others would treat them. Example items of social ostracism were, “If people know that I have schizophrenia, my interpersonal relationship will be damaged” or “If people know that I have schizophrenia, my reputation will be harmed.” The second subscale (marital preclusion) assessed hypothetical concerns regarding marital obstacles that may occur due to schizophrenia. Example items of marital preclusion were, “Because I have schizophrenia, I believe that nobody is willing to marry me” or “Because I have schizophrenia, I believe that the parents of my partner will oppose our marriage.” The third subscale (self-deprecation) examined the respondents’ negative self-image under the premise that they had schizophrenia. Example items of self-deprecation were, “I am a weakling because I have schizophrenia” or “Because I have schizophrenia, I am ashamed of myself.” Items were scored on a four-point Likert scale ranging from 1 (completely disagree) to 4 (completely agree). A composite measure of self-stigma is derived by totaling the sum of all statements (range: 25–100). The higher the score, the more self-stigma demonstrated. The validation study of the Perceived Psychiatric Stigma Scale reported that Cronbach’s α was 0.94 and test–retest reliability with one-week interval was 0.90 [21].

#### 2.3.3. Social Distance

We used a modified version of the Bogardus Social Distance Scale [22] to assess social distance toward individuals with schizophrenia. Social distance was first conceptualized by Park and Burgess in the 1920s as a measure of attitudes toward certain social topics among individuals or groups. Differences in social distance exist between ethnic groups, sexes, social classes, careers, religions, and countries [23]. According to experts’ opinions, we chose seven questions from the original scale that reflected students’ daily lives. Different distances represented different levels of intimacy. For example, “I am willing to stay in the same city with the patient” and “I am willing to marry a patient with schizophrenia” represented the greatest and smallest social distances, respectively. To compare social distance before and after the renaming, one column used the old name and the other used the new name, with the questions remaining identical. We used a Guttman scale with the categories of disagree and agree in this questionnaire. Guttman scale is a cumulative scale designed so that agreement with higher-level responses assumes agreement with all lower-level responses. A total score ranges from 0 to 7, with higher scores reflecting higher levels of social distance.

### 2.4. Statistical Analysis

From the demographic data, descriptive statistics (means ± standard deviations, frequencies, and percentages) were extracted. We categorized the participants into two groups, namely the first-year student group and the fourth-year student group. Between-group differences in continuous variables were examined using Student’s *t* test, whereas those among categorical variables were evaluated using Fisher’s exact test.

The dependent variables were the differences in the stigma scale scores between old and new name of schizophrenia. The Shapiro–Wilk method was used for normality test. The independent variable selection (e.g., demographic characteristics and previous contact experience) in the multiple liner regression model was based on the results of exploratory univariate linear regressions. Only variables that were associated with dependent variables in the univariate analyses (with *p* < 0.05) were included as independent variables in a subsequent multiple linear regression.

All statistical analyses were performed using IBM SPSS Statistics for Windows, version 19.0 (IBM Corp., Armonk, NY, USA). A *p* value lower than 0.05 was considered as statistically significant.

## 3. Results

Of the 120 questionnaires distributed, 102 were returned. After excluding incomplete responses, 99 questionnaires remained; the final sample comprised 19 men and 80 women (Table 1). No significant differences in demographic characteristics (except age) or in previous contact experience with individuals with mental disorders were observed between the first- and fourth-year students. The first-year students (mean ± standard deviation; 19.8 ± 2.3 years) were significantly younger than the fourth-year students (22.7 ± 0.8 years) (*p* < 0.001).

**Figure 1:**
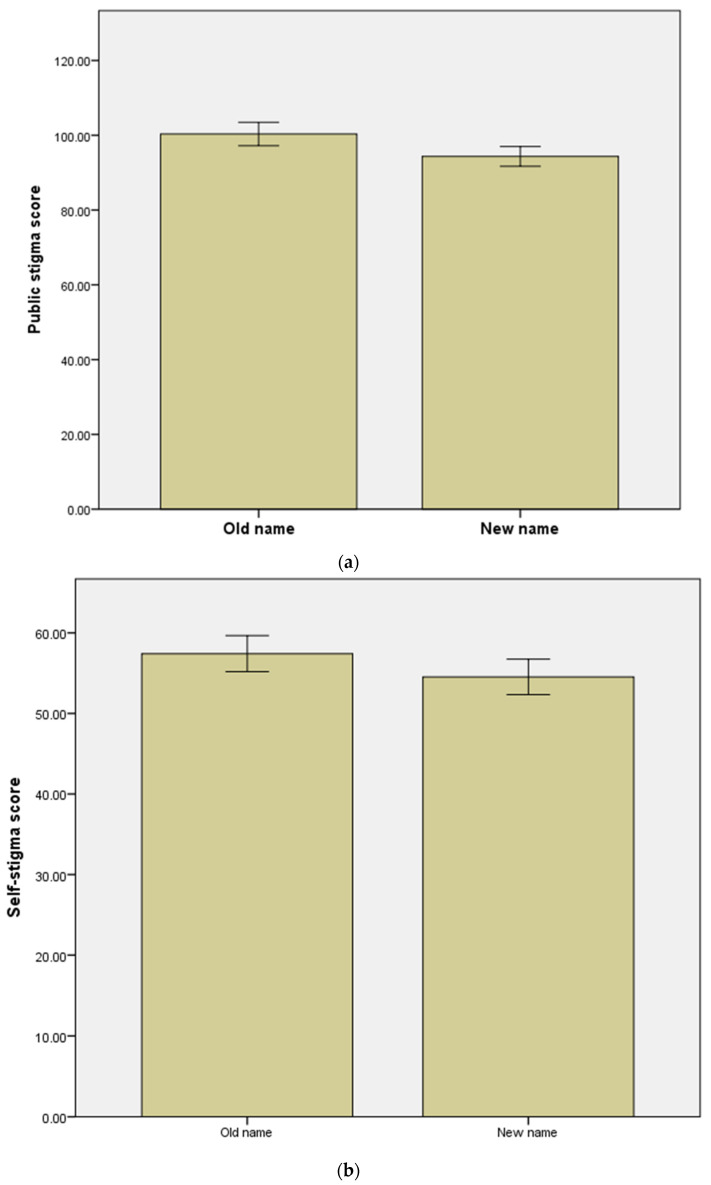

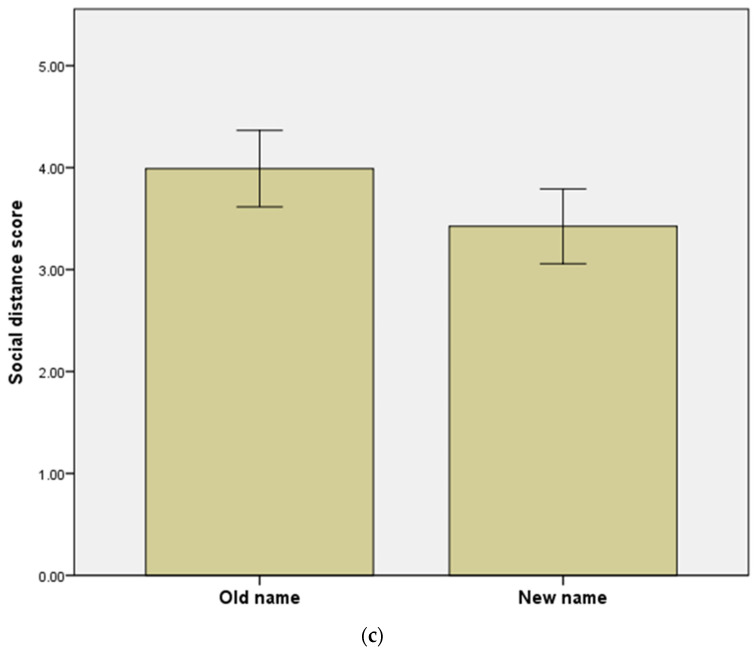
Effect of renaming schizophrenia on stigma parameters. (**a**) Public stigma; (**b**) self-stigma; (**c**) social distance.

Overall, significant reductions in public stigma, self-stigma, and social distance after renaming were noted in all participants (Figure 1) (Table 2). The old name for schizophrenia corresponded to significantly higher scores than the new name on the three self-stigma subscales (social ostracism, marital preclusion, and self-deprecation) among all participants.

When categorized participants into the first-year group and the fourth-year group, the new name of schizophrenia had considerably lower scores in public stigma, self-stigma, and social distance than the old name of schizophrenia in both groups. Among the subscale of self-stigma, only scores on the self-deprecation subscale remained similar among the first- and fourth-year students after the renaming. Compared with the first-year students, the fourth-year students scored significantly higher on public stigma and self-stigma in the old name and new name of schizophrenia, respectively (Table 2).

The correlations between the changes in the stigma scores, demographic characteristics, and previous experiences of contact with people diagnosed with mental illness were presented in Table 3. The change in the score of public stigma was correlated with having classmates with a mental illness (β = −5.33, *p* = 0.021) and having relatives with a mental illness (β = −5.12, *p* = 0.040) in univariate linear regressions.

To identify association with stigma outcomes, multiple regression analysis was performed, with changes in stigma outcomes as the dependent variable. The independent variables constituted various measures for which considerable correlations had been observed in the univariate linear regression analysis. With changes in public stigma as the dependent variable, multiple regression analysis revealed that having classmates with a mental illness (β = −0.315, *p* = 0.013) and having relatives with a mental illness (β = −0.225, *p* = 0.031) were significant associated with it. No variable was associated with the changes in self-stigma or social distance in multiple regression analyses.

## 4. Discussion

The results demonstrated that renaming mitigated the stigmatization of individuals with schizophrenia by nursing students in Taiwan. We found that the scores of the new name of schizophrenia in the assessments of public stigma, self-stigma, and social distance were lower than those corresponding to the old name of schizophrenia. However, the fourth-year nursing students scored significantly higher in the assessment of public stigma and self-stigma than the first-year nursing students did.

The public stigma scores corresponding to the new name were significantly lower than those corresponding to the old name. In other words, the renaming was associated with reducing public stigma toward schizophrenia in nursing students. This finding is consistent with that of a review article, which revealed an association between the renaming of schizophrenia and improvements in attitudes toward individuals with this disorder [10].

The self-stigma scores decreased significantly after the renaming on all three of the subscales (social ostracism, marital preclusion, and self-deprecation) in all participants, with marital preclusion having the most notable results. In contrast with marriage in the Western countries, Taiwanese marriage often extends beyond the union of a couple to the union of their families. The traditional concept holds that marriages should ideally be between two families of equal social ranking and status. Many people are unwilling to accept people with mental disorders into their families, and patients often limit themselves in this respect. Han and Chen [21] asserted that marital preclusion is a key issue in the stigmatization of mental disorders in Taiwan. In the present study, renaming schizophrenia reduced self-stigma, especially with regard to marital preclusion.

As for social distance, the new name corresponded to a closer social distance than did the old name. The more individuals with schizophrenia are perceived as dangerous and violent, the greater is the social distance the general population wishes to keep from this patient population [24,25]. Considerably lower scores were noted on questions about danger and fear when the new name was used than when the old name was used. Thus, the new name reduced the feelings of fear and danger associated with schizophrenia, thereby reducing the social distance.

Regarding self-deprecation, no significant differences between the old and new names were observed in the first- and fourth-year students, suggesting that the renaming failed to mitigate the stereotyped concept of functional impairment in schizophrenia among the participants. Both before and after renaming, the respondents held a negative self-image in imagining themselves as individuals with schizophrenia.

An unexpected finding was that the fourth-year students’ scores on public stigma and self-stigma were significantly higher than those of the first-year students. One possible explanation is that those differences are due to age/generation effects. Another possible explanation for the stigmatization of schizophrenia among nursing students is the students’ experiences of the emotional distress or disturbing behaviors exhibited by people with schizophrenia [26]. Notably, the fourth-year students have already completed both classroom education and a clinical internship on psychiatry. Our results suggested that knowledge and clinical contact with patients might negatively affect these students’ attitudes toward schizophrenia. Hawthorne et al. [27] observed a J curve between clinical experience and stigma toward mental disorders; that is, stigma was initially exacerbated by low clinical exposure to patients with mental disorders but was subsequently mitigated with increasing exposure. On the basis of our findings, we suggest that appropriate psychiatric nursing education programs are warranted to provide accurate information about schizophrenia, instruction by qualified tutors, and exposure, not only to patients with schizophrenia in acute psychotic state in hospital settings but also to recovered patients in the community.

In the present study, we found that having relatives and classmates with a mental illness both affected public stigma. Our results supported the contact hypothesis, which posits that more contact with people with mental disorders should promote positive attitudes toward them, and confirmed the observations from relevant studies [16,27,28].

## 5. Limitations and Future Directions

This study has several methodological limitations that constrain the generalizability of the findings. First, the study participants were nursing students at Taipei Medical University, and the potential for selection bias should be considered. Second, this study was a cross-sectional survey that could not determine any causal associations. Third, because the participants were all nursing students, the findings may not reflect the attitudes of the general population. Fourth, the small sample size may limit the generalization of our study. Fifth, the modified Corrigan’s attribution questionnaire and the revised modified Bogardus’s social distance scale were not fully validated in Taiwan. Adaptations of existing Western-developed stigma measures to Taiwanese warrant further investigations.

Further studies in this field are recommended. It is desirable that the limitations of our study are taken into consideration. First, studies with large sample sizes and participants from diverse backgrounds are warranted. Second, prospective studies with multiple follow-up evaluation sessions are warranted to monitor the changes in attitude and behavior. Third, the development of appropriate psychiatry education for nursing students to against stigma toward people with mental illness is important.

## 6. Conclusions

In this investigation of nursing students, the renaming was found to shorten social distance from people with schizophrenia as well as reduce both public stigma and self-stigma toward schizophrenia. The findings indicate that renaming might be an effective strategy for reducing nursing students’ stigmatization toward schizophrenia. Appropriate psychiatric nursing education programs should be established to provide accurate information about schizophrenia, instruction by qualified tutors, as well as exposure—not only to patients with schizophrenia in acute psychotic state in hospital settings but also to recovered patients in the community. Further studies with longitudinal design, participants from diverse backgrounds, and larger sample sizes to investigate the effect of renaming on the stigma toward schizophrenia are warranted.

## Figures and Tables

**Table 1:** Demographic characteristics.

Characteristics	Total	First-Year	Fourth-Year	*p* Value *
Sex				0.228
Female	80	32	48	
Male	19	10	9	
Age	21.4 ± 2.2	19.8 ± 2.3	22.7 ± 0.8	<0.001
Religion				0.161
No	55	28	27	
Buddhism/Taoism	31	10	21	
Christian/Catholicism	13	4	9	
Relative with mental illness				0.892
No	70	30	40	
Yes	29	12	17	
Friend with mental illness				0.115
No	72	34	38	
Yes	27	8	19	
Classmate with mental illness				
No	59	28	31	0.218
Yes	40	14	26	

* comparison between the first-year group and the fourth-year group.

**Table 2:** The effect of renaming.

	Total (*n* = 99)		First-Year (*n* = 42)		Fourth-Year (*n* = 57)	
	Old Name	New Name	Old Name	New Name	Old Name	New Name
**Public stigma**	100.3 ± 15.7 ***	94.3 ± 13.3	95.2 ± 15.1 **^,##^	88.7 ± 13.6 ^###^	104.2 ± 15.1 ***	98.5 ± 11.5
**Self-stigma**	57.4 ± 11.2 ***	54.5 ± 11.1	53.2 ± 9.8 **^,###^	50.1 ± 10.2 ^###^	60.5± 11.2 **	57.8 ± 10.6
**Social ostracism**	23.2 ± 5.0 ***	22.0 ± 4.9	20.9± 4.3 **^,###^	19.8 ± 4.3 ^###^	24.9 ± 4.9 **	23.7 ± 4.6
**Marital preclusion**	21.1 ± 4.2 ***	19.9 ± 4.2	20.0± 4.2 ***^,#^	18.6 ± 4.3 ^##^	22.0 ± 4.1 **	20.8 ± 3.9
**Self-deprecation**	13.1 ± 3.1 *	12.6 ± 3.1	12.3 ± 2.6 ^#^	11.7 ± 2.9 ^#^	13.6 ± 3.3	13.3 ± 3.2
**Social distance**	4.0 ± 1.9 ***	3.4 ± 1.8	4.0 ± 1.8 *	3.5 ± 1.9	4.0± 1.9 **	3.4 ± 1.8

*** *p* < 0.001, ** *p* < 0.01, * *p* < 0.05 within-group comparison. ^###^ *p* < 0.001, ^##^ *p* < 0.01, ^#^ *p* < 0.05 between-group comparison.

**Table 3:** Results of univariate analyses on the changes in the scores of stigma scale.

	Changes in the Scores of Public Stigma		Changes in the Scores of Self-Stigma		Changes in the Scores of Social Distance	
	B (95% CI)	*p* Value	B (95% CI)	*p* Value	B (95% CI)	*p* Value
The fourth year student	−0.74 (−5.33 to 3.84)	0.748	−0.40 (−3.01 to 2.26)	0.766	0.07 (−0.53 to 0.68)	0.239
Female sex	−0.65 (−6.41 to 5.11)	0.823	−0.14 (−3.47 to 3.20)	0.935	0.05 (−0.71 to 0.81)	0.899
Age	−0.20 (−1.27 to 0.86)	0.706	−0.27 (−0.89 to 0.34)	0.381	0.01 (−0.14 to 0.14)	0.958
With religion belief	2.62 (−1.92 to 7.15)	0.255	1.10 (−1.53 to 3.73)	0.409	0.17 (−0.43 to 0.77)	0.578
Relative with mental illness	−5.12(−9.99 to −0.24)	0.040	0.45 (−2.43 to 3.33)	0.758	−0.12 (−0.77 to 0.54)	0.723
Friend with mental illness	0.82 (−4.28 to 5.91)	0.751	1.43 (−1.51 to 4.36)	0.965	0.50 (−0.17 to 1.16)	0.140
Classmate with mental illness	−5.33 (−9.83 to −0.83)	0.021	1.61 (−1.04 to 4.27)	0.231	0.56 (−0.04 to 1.16)	0.07

## Data Availability

The datasets generated for this study are available on request to the corresponding author.
